# Cannabinoids infused mouthwash products are as effective as chlorhexidine on inhibition of total-culturable bacterial content in dental plaque samples

**DOI:** 10.1186/s42238-020-00027-z

**Published:** 2020-06-23

**Authors:** Kumar Vasudevan, Veronica Stahl

**Affiliations:** CannIBite, Mortsel, 2640 Antwerp, Belgium

**Keywords:** Cannabinoids, CBD, CBG, Oral care, Dental plaque, Oral health, Mouthwash

## Abstract

**Background:**

Dental plaque is a global health problem affecting people of various age groups. Cannabinoids are gaining enormous research attention due to its beneficial properties for various applications. A preliminary observation on antimicrobial property of cannabinoids against dental plaque bacteria has been reported recently. As a follow-up research, here we report the in vitro evaluation of cannabinoids infused mouthwash products against total culturable (aerobic) bacterial content from dental plaque samples.

**Methods:**

We tested two cannabinoid-infused mouthwash products containing cannabidiol (CBD) and cannabigerol (CBG) respectively (each mouthwash containing < 1% cannabinoid by weight) in vitro against total-culturable bacteria from dental plaque samples collected from 72 adults aged between 18 and 83 years. The participants were grouped on the basis of Dutch periodontal screening index (DPSI) score. To compare the efficacy of our products, we included two most commonly available products over the counter (Product A and Product B) to represent commercially available mouthwash products and the gold standard chlorhexidine digluconate 0.2% as a positive control. The product A represents mouthwash containing essential oils and alcohol, and Product B represents alcohol-free mouthwash that contains fluoride. All the mouthwash products were evaluated directly as such without any dilution through disc diffusion and agar well diffusion approaches and the diameter of zone of inhibition was measured. The limitation in methodology was that, the samples were open-label and the person who performed the manual measurements was unblind to test and control products used.

**Results:**

On average, the cannabinoids infused mouthwash products showed the similar bactericidal efficacy as that of chlorhexidine 0.2%. Both chlorhexidine 0.2% and cannabinoids infused mouthwash products were effective against all the samples tested. Product A did not show any significant antimicrobial activity in any of the samples tested, except that a very marginal inhibition with a zone of 7-8 mm was observed only in 9 samples. Product B did not show any detectable inhibition zone at all in any of the samples tested. The ranges of zones of inhibition (and their average) were 8–25 mm (18.1 mm) for CBD-mouthwash, 8–25 mm (17.7 mm) for CBG-mouthwash; 12–25 mm (16.8 mm) for chlorhexidine 0.2%; 0–8 mm (0.1 mm) for Product A; and 0 mm for Product B. Although the difference in performance was slightly higher than chlorhexidine in both the cases, the difference was statistically significant for CBD-mouthwash and near significant for CBG-mouthwash. No significant difference was observed between CBD- and CBG-mouthwash. No significant difference in performance was found between DPSI score groups for any of the product tested. To our knowledge this is the first report on such efficient mouthwash product with natural key ingredients including cannabinoids and without any kind of fluoride or alcohol.

**Conclusions:**

Our in vitro results demonstrate the potential of cannabinoids in developing efficient and safer mouthwash products and next generation oral care products without fluoride and alcohol.

## Background

Dental plaque is a complex biofilm composed of diverse species of microbial community accumulated on the surface of the teeth, eventually contributing to the development of caries and periodontal disease. The development of dental plaque involves adherence of primary colonizing bacterial species to the enamel salivary pellicle followed by secondary colonizers through interspecies interactions and communications (Rosan and Lamont 2000). Various factors may influence dental health including diet and lifestyle (Scardina and Messina 2012; Hasselkvist et al. 2014). Dental hygiene requires proper regular care with suitable products and post-brushing rinsing has been reported to be effective in reducing the dental plaque and gingivitis (Stoeken et al. 2007; Prasad et al. 2016).

Some of the commercial mouthwash products available over the counter (OTC) are reported to be poorly effective against pure isolates of few bacterial species, however chlorhexidine is the most effective mouthwash in controlling dental plaque (Zheng and Wang 2011; Müller et al. 2017). Chlorhexidine is often referred as “the gold standard” in dentistry and has been reported to be very effective in reducing the dental plaque, gingivitis and biofilm formation; however produce the unpleasant side effects of tooth discoloration/staining and calcium buildup (Varghese et al. 2019). Similarly, systematic review reports have also pointed out tooth surface discoloration as a potential negative side effect of chlorhexidine (Slot et al. 2014; Richards 2015). Addition of antidiscoloration system with chlorhexidine might actually impair the actual function of chlorhexidine (Li et al. 2014; Guerra et al. 2019). Several systematic reviews have reported that some herbal mouthwashes including *Aloe vera* and neem as potential alternatives for chlorhexidine (Dhingra and Vandana 2017; Al-Maweri et al. 2020). However, systematic reviews on herbal mouthrinses have reported herbal mouthrinses as beneficial or have the potential to equate chlorhexidine, the evidence is insufficient and more comprehensive and controlled studies are need to be done to scientifically validate the herbal products (Chen et al. 2014; Manipal et al. 2016).

Cannabinoids are phytochemicals / secondary metabolites naturally produced by cannabis plant (*Cannabis sativa* L.) which include some psychoactive compounds such as Δ9-tetrahydrocannabinol (Δ9-THC) and various non-psychoactive compounds such as cannabichromene (CBC), cannabidiol (CBD), cannabigerol (CBG) and cannabinol (CBN) (Andre et al. 2016; Pellati et al. 2018). Cannabinoids are reported to have antibacterial activity against several gram-positive as well as gram-negative bacterial species (Wasim et al. 1995; Appendino et al. 2008; Sarmadyan et al. 2014; Kosgodage et al. 2019). An interesting detailed molecular study reported that synthetic cannabinoid interferes in AI-2 quorum sensing signal cascade in *Vibrio harveyi* (Soni et al. 2015). The periodontal pathogenic bacteria are also reported to possess AI-2 quorum sensing system to communicate and to regulate various function including biofilm formation, stress response and virulence factor expression (Plančak et al. 2015; Basavaraju et al. 2016).

The combinatorial ability of cannabinoids as antimicrobial agent together with ability to interfere in AI-2 quorum sensing signal cascade makes cannabinoids a perfect candidate to apply in dental care. We previously reported our preliminary observatory report on efficiency of cannabinoids in reducing bacterial content in dental plaque (Stahl and Vasudevan 2020). As a follow up research, we evaluated the efficiency of cannabinoids infused mouthwash products developed by CannIBite, in comparison with other commonly available commercial mouthwash products and the gold standard chlorhexidine. We evaluated the products with open-label (unblind) under in vitro conditions. Here we report the efficiency analysis of bactericidal activity of cannabinoids infused mouthwash products against total-culturable bacterial content from dental plaque samples.

## Methods

### Study population

A randomized controlled trial was conducted between October 2019 and March 2020 in Belgium. The study protocol was reviewed and cleared by the Ethics Committee of the Institutional Review Board (AZ Groeninge Kortrijk, Belgium). The study protocol and the purpose were explained orally to each participant. Oral and signed consent from each participant was obtained before the start of the study.

A total of 72 adults (38 women and 34 men), aged between 18 and 83 were chosen for the study from Euro-Dent clinic, Mortsel 2640, Belgium. For convenience, the study sample was chosen among the clinic patients who are eligible (criteria described below) and agreed to participate in the study with consent. Our study population represent all age group adults i.e., young (18–35; *n* = 33), middle aged (36–55; *n* = 28) and elderly (56–90; *n* = 11). The chosen 72 adults satisfied the following selection criteria for the study: (a) presence of a minimum number of teeth (seven), including one molar, (b) absence of dentures, (c) no recent history of antimicrobial therapy or other drug therapy, including immunosuppressive, and (d) no history of diabetes.

The periodontal score of the participants were scored on the basis of Dutch periodontal screening index (DPSI) as follows: 0, perfect gum and no bleeding; 1, inflammation and bleeding of gum (gingivitis); 2, conditions of category 1 and chalk hardened dental plaque; (− 3), conditions of category 2 with bone involvement (periodontitis); (+ 3) conditions of (− 3) with recessions of gum and root exposure; and 4, conditions of category (+ 3) with severe bone resorption and high tooth mobility. The number of individuals with DPSI score 0, 1 and 4 were extremely rare in Euro-Dent clinic during our study period, therefore we did not involve these three DPSI score categories in our study. Our study population involve 72 participants in total, which include DPSI score 2 (50 participants); DPSI score + 3 (10 participants) and DPSI score − 3 (12 participants). The chosen 72 participants were not taking any special treatments for dental plaque before or during the course of study.

### Equipment and chemicals

The disposable sampling microbrush applicators were purchased from Microbrush International (Ireland). The plastic consumables like microtubes, petriplates, ready-to-use media of LB agar plates and DEV-Nutrient agar plates (90 mm) and paper discs were purchased from VWR International (Belgium). Product A, Product B and chlorhexidine 0.2% were purchased from local supermarket/medical store in Belgium. Pure isolates of CBD and CBG crystalline powders were purchased from PharmaHemp (Slovenia). The Synbiosis-aCOLyte 3 HD colony counter and Memmert incubator were purchased from Wilten Instrumenten (Netherlands). The sterile plate spreaders, 50 ml sterile conical tubes and Biosan orbital shaker-incubator were purchased from NOVOLAB (Belgium).

### Dental plaque sampling

Prior to plaque sampling, saliva on the tooth surface was removed by water spray, and the sampling target area was dried with cotton. Plaque samples were collected from interdental spaces using disposable microbrush applicator and immediately dispensed into a 2 ml microtube containing 1 ml of phosphate buffer saline (PBS) and was processed for in vitro assay within 24 h from sampling.

### Test material-mouthwash products

The test products i.e., CannIBite mouthwash products contain herbal ingredients including cannabinoids, wintergreen oil and stevia extract. Two test mouthwash (MW) products were used in this study i.e., CBD-MW containing (< 1% by weight) cannabidiol (CBD) and CBG-MW containing (< 1% by weight) cannabigerol (CBG). Most commonly available OTC mouthwashes, designated hereafter as Product A and Product B mouthwash were also used in this study to compare the efficacy of our test products. Product A represents alcohol-containing mouthwash with essential oils such as thymol, eucalyptol, and menthol as active ingredients. Product B represents alcohol-free mouthwash containing fluoride and potassium nitrate as active ingredients. In addition, the medical grade mouthwash containing 0.2% chlorhexidine (the so-called gold standard) was used as positive control. All the mouthwash products used in this study were directly used for in vitro assay without any dilutions.

### In vitro assay

The dental plaque sample was mixed well using vortex and an aliquot of 100 μl of dental plaque sample was spread on the surface of LB agar plate or DEV-Nutrient agar plate using sterile spreader. For agar well diffusion method, agar well of 4 mm diameter were made (on plate pre-inoculated with dental plaque sample) using sterile agar well borer and 30 μl of undiluted MW was added to the well. In case of disc diffusion method, sterile paper disc of 5 mm diameter was placed on the surface (on plate pre-inoculated with dental plaque sample) and 15 μl of undiluted MW was gently added to the disc. To minimize the bias in data, we included three technical replicates for each sample. Each sample assay was performed in triplicates.

The petri dishes were sealed with thin layer parafilm (to reduce the evaporation of test products) and incubated at 37 °C for 36 h. After incubation, the diameter of zone of inhibition was measured manually using caliper. The pictures of plates were taken using automated colony counter. Zone of inhibition was not recorded using colony counter due to varying colony size, density, color and texture (within and between samples) because of the presence of multiple species of bacteria. Reducing the colony size threshold beyond a limit (< 0.2 mm) in colony counter leads to false reading. Moreover, the paper discs on the plate interfere with the contrast of colony vs. media background and affects the reading by colony counter. However the zone was clearly visible when the plate was observed in front of bright light source such as lamp and was easy to measure manually.

For minimum inhibitory concentration (MIC) assay, the test products were serially diluted with sterile LB broth media from 0 to 11 dilutions (0 represents undiluted test product) in 50 mL sterile conical tubes. After inoculating equal amount of dental plaque bacteria (fresh overnight culture pre-prepared a day before) in each tube containing 2 mL of respective serially diluted media, the samples were incubated in orbital shaker-incubator at 37 °C for 24 h. For comparison, we also tested the MIC assay for Product A, Product B and CHX 0.2%.

The liquid culture turbidity (for MIC) and plate readings (for zone of inhibition) were measured by the same person (unblind to test and control products used) who performed the assay, which was a major limitation in methodology as the samples were open-label.

### Statistical analysis

The in vitro experiments were conducted in triplicates. The average values of zone of inhibition for each of the samples were calculated in Microsoft Excel (Table S1; and raw data provided as Table S2). The average values were used to represent in graphs and table. Student’s unpaired t-test was performed to compare the results between DPSI score groups for each test product (Table S3). Student’s unpaired t-test was also performed to compare the results between products irrespective of DPSI score grouping (Table S3).

## Results

### Cannabinoid infused mouthwash products perform equal or better than that of chlorhexidine 0.2%

**Fig. 1:**
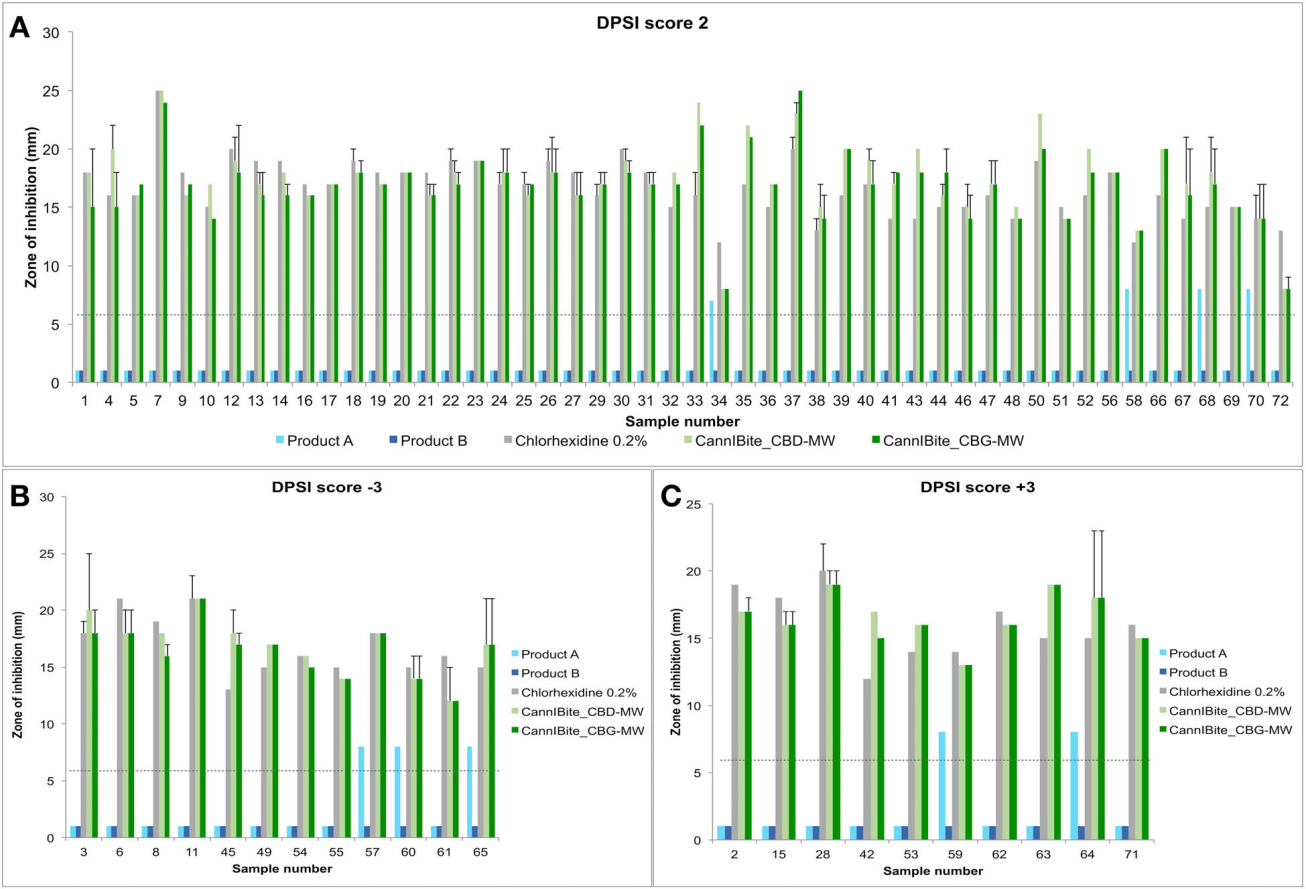
Zone of inhibition data measured in mm (diameter of zone). **a** DPSI score 2 group; **b** DPSI score − 3 group; **c** DPSI score + 3 group. The reference line represents the size of paper disc used (6 mm). The values below reference line for Product A and Product B represents zero inhibition, and a false minimum value of 1 was given to visualize in the graph. The positive error bars represent secondary inhibition zone (diameter in mm)

**Fig. 2:**
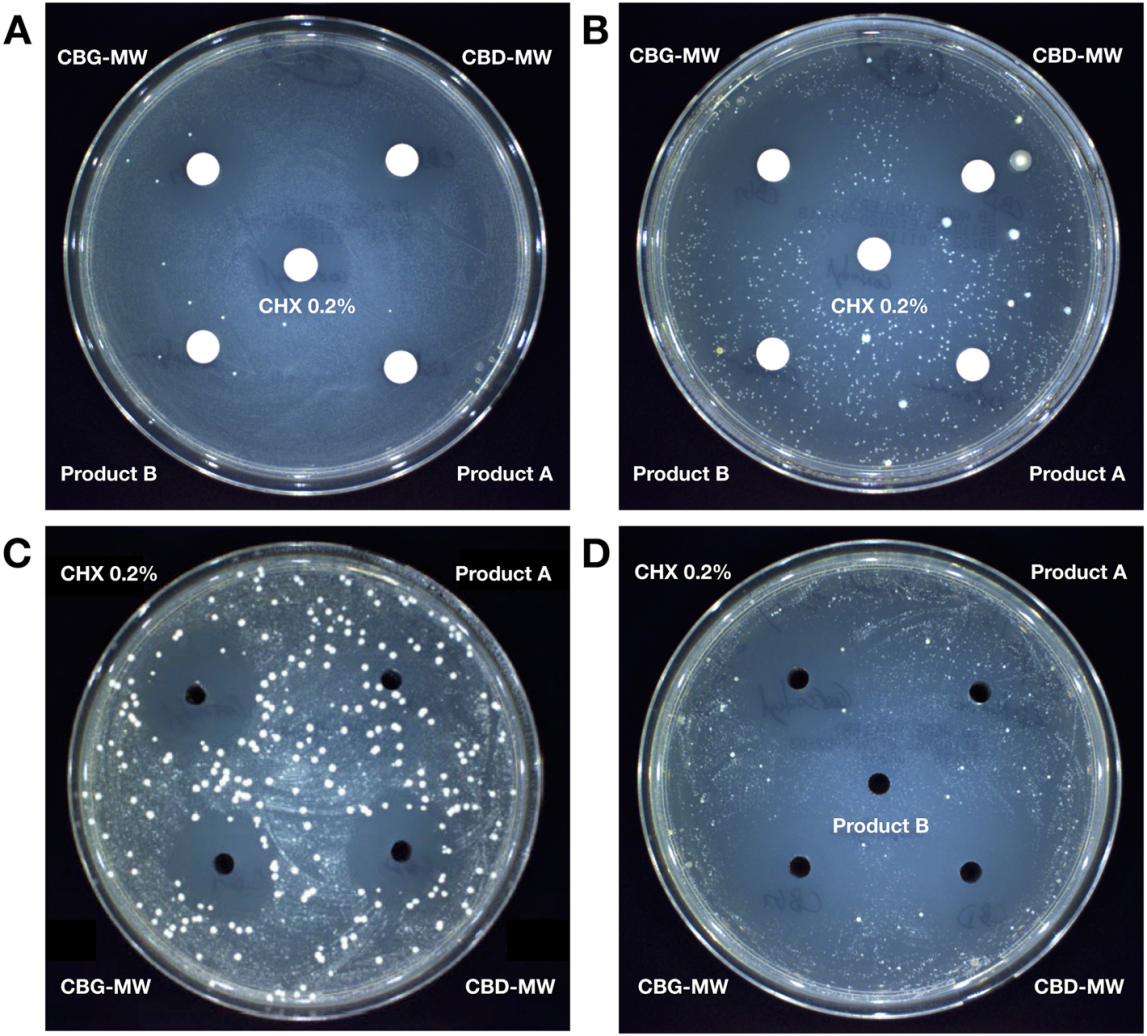
Microbiological assay of mouthwash products against total-culturable aerobic bacterial content from dental plaque samples. Some examples from disk diffusion method (**a**, **b**) and agar well diffusion method (**c**, **d**) are shown. CHX0.2% = chlorhexidine 0.2%. Disk = 6 mm; agar well = 4 mm

**Fig. 3:**
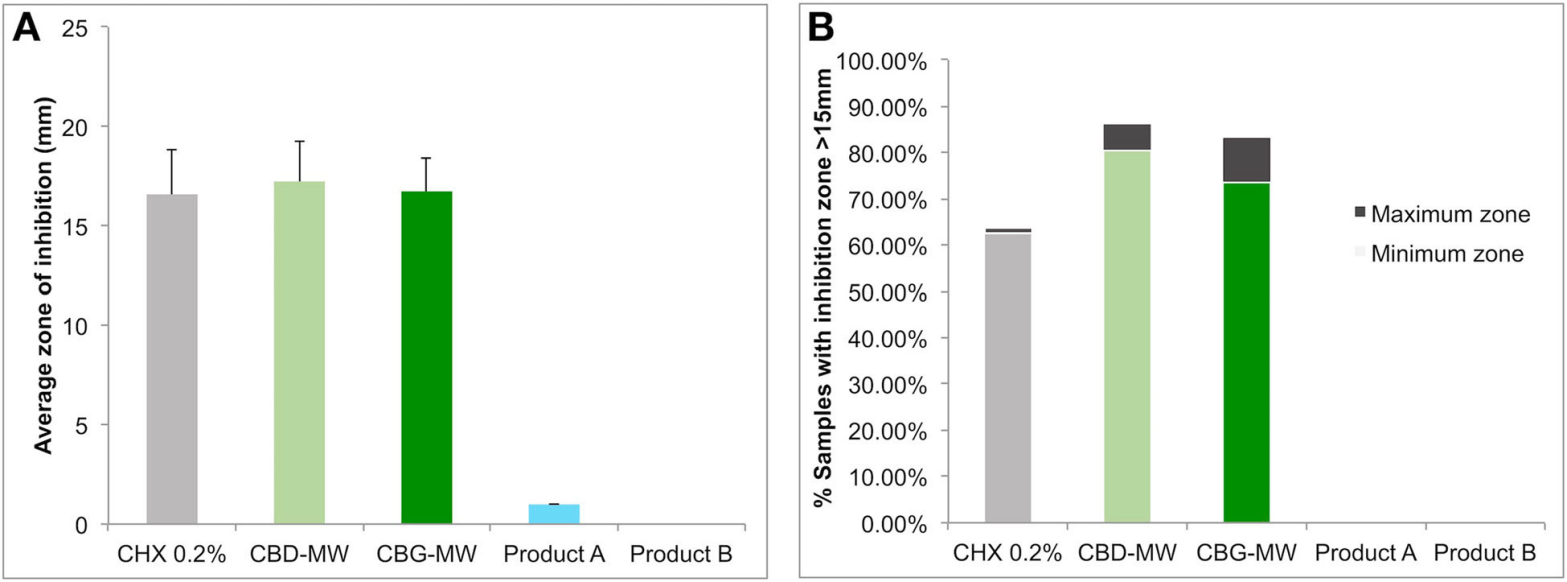
Panel A, the averages of inhibition zones of all samples combining all DPSI score groups. The positive error bars represent secondary inhibition zone. Panel B, the percentage of samples with inhibition zones greater than 15 mm. The percentages including and excluding the secondary zones are highlighted separately as minimum and maximum zones

Chlorhexidine 0.2% (CHX 0.2%) showed consistent bacterial growth inhibition with clear zone of inhibition on all the samples tested in this study (Fig. 1, Fig. 2). On average, both CBD-MW and CBG-MW showed similar efficiency as that of CHX 0.2%, the gold standard (Fig. 1, Fig. 2, Table S1, Table S2). In some samples, CBD-MW and/or CBG-MW showed better efficiency of bacterial growth inhibition than that of CHX 0.2% (Fig. 1, Table S1). Both Product A and Product B mouthwashes did not form any inhibition zone at all except Product A, which showed very minimal inhibition zone only in 9 samples (Table S1). The range of zones of inhibition was 8–25 mm for both CBD- and CBG-mouthwash, however the average was 18.1 mm and 17.7 mm for CBD-mouthwash and CBG-mouthwash respectively (Table S1). In case of CHX 0.2%, the range was 12–25 mm with an average of 16.8 mm (Table S1, Fig. 3). The performance of CBD-MW and CBG-MW were much better than CHX 0.2% in several samples based on the percentage of samples with inhibition zone greater than 15 mm being 63.8% in CHX 0.2, 86.1% in CBD-MW and 83.3% in CBG-MW as the maximum values (Fig. 3, Table S1).

### Comparison of growth media and assay methods

To test the effect of growth media on bacterial inhibition, the same assays were performed on LB and DEV-Nutrient agar plates and no significant difference in level of zone of inhibitions were observed for any of the mouthwash tested (Fig. S1). However, slight difference in density of bacterial colonies was observed, which might indicate the effect of media composition on bacterial growth. Since the effect of growth media did not significantly affect the level of zone of inhibition, the comparison of assay on LB and DEV-Nutrient agar was not performed for all the samples.

Similarly, we compared the zone of inhibition in agar well diffusion and disc diffusion methods. The trend in level of zone of inhibition for each tested product was similar (Fig. 2) irrespective of disc or agar-well diffusion methods.

### Determination of minimum inhibitory concentration

In CBD-MW and CBG-MW, bacterial growth was observed at 8th dilution; in Product A and Product B, growth was observed from 3rd dilution; whereas in CHX0.2%, growth was observed at 10th dilution (Fig. S2). The MIC was calculated based on the formula, MIC = lowest concentration of product inhibiting growth + highest concentration of product allowing growth / 2. By considering the final formulation/product as 100% and on the basis of v/v calculation using the above formula, the MIC values were calculated as 0.24% for CHX 0.2%; 0.58% for CBD-MW and CBG-MW; and 18.75% for Product A and Product B. Based on these results, the MIC value of CBD-MW and CBG-MW were closer but slightly (2.37 times) higher than that of positive control CHX 0.2%, whereas the MIC value of Product A and Product B were much (78.12 times) higher than that of CHX 0.2%.

### Second layer of partial inhibition zone was observed in some samples

**Fig. 4:**
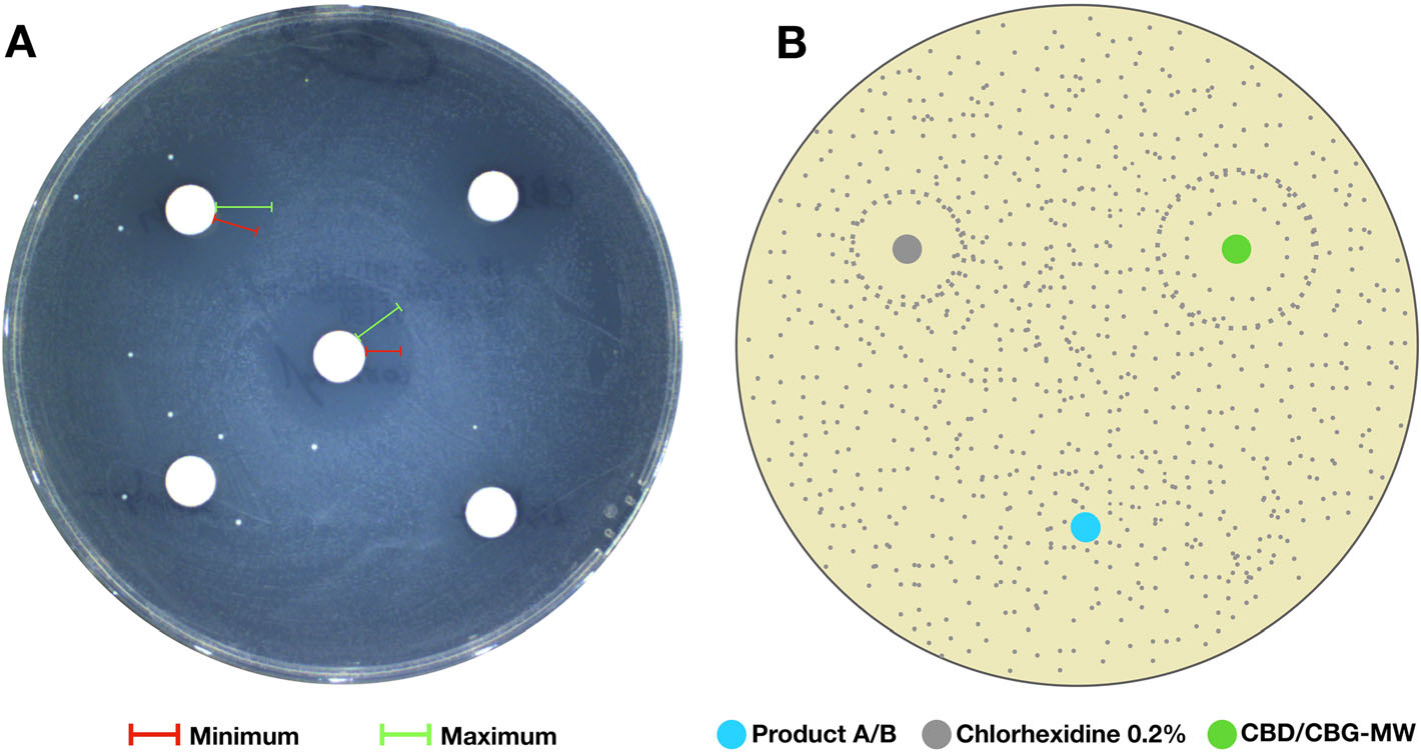
**a** Example plate with minimum and maximum inhibition zones. **b** Schematic representation of single and double inhibition zones

CHX 0.2% showed clear and sharp zone of inhibition in most of the samples tested. In case of CBD-MW and CBG-MW, the zone of inhibition was clear and sharp in some samples, whereas in some samples there were two layers of inhibition zones (Fig. 1, Fig. 3, Fig. 4). In this case, the immediate zone was a clear inhibition zone and the secondary zone was a partial inhibition zone (Fig. 4a). Such observation suggests the possibilities of selective inhibition towards some species of bacteria over the other beyond a threshold (the first zone). Since it is difficult to clearly visualize the two zones in picture, a schematic representation of this observation is showed in Fig. 4b. In Table S1, the samples with minimum and maximum values represent such two-layers of inhibitory zones. The minimum values represent the clear immediate inhibition zone (almost complete absence of any bacterial colony) and the maximum values represent the secondary inhibition zone (partial inhibition with very less colony density than the rest of the plate). The samples with single data entry (minimum values alone) represent the clear and sharp single zone of inhibition (Table S1).

Product B did not form any detectable inhibition zone at all in any of the samples studied. Bacterial colonies were observed immediately next to the paper disc or agar well (Fig. 2, Fig. 4). Similarly, Product A did not form any detectable inhibition zone except for few samples, in which only a minimal inhibition was observed (Fig. 1, Table S1).

### Comparison of products and performance among DPSI score, gender and age groups

By comparative statistical analysis of performance between the products irrespective of DPSI score grouping, it was observed that CBD-MW was significantly (*P*-value < 0.05) better than CHX 0.2% (however the average quantitative difference was minor). Similarly, CBG-MW was slightly better than CHX 0.2% with a near significant (*P*-value < 0.06) difference (Table S3). There was no significant difference (*P*-value > 0.3) between CBD-MW and CBG-MW. To understand if the test products work better only on a particular DPSI score category, a comparative analysis was performed. Based on comparative statistical analysis between DPSI score groups for the same product, no significant difference (*P*-value > 0.3) was observed for any of the products tested (Table S3). Similarly, no statistical conclusion could be drawn (*P*-value > 0.1) on product performance with respect to age or gender (Table S3). Since none of the products tested showed variable performance based on age, gender or DPSI score categories, no additional statistical analysis was performed. Therefore, irrespective of age, gender and DPSI score, on average, both CBD-MW and CBG-MW showed similar or better efficiency than that of positive control (CHX 0.2%) used in this study.

## Discussion

In this study we demonstrated the efficiency of cannabinoids infused mouthwash products against dental plaque bacteria. Cannabinoids, the secondary metabolites produced by *Cannabis sativa* L. plant are gaining enormous research interest in the recent past due to its various beneficial properties in the field of pharmaceutical and cosmetic industry. In addition, the antibacterial properties of cannabis essential oils and cannabinoids are also being reported (Appendino et al. 2008; Iseppi et al. 2019) including antibacterial activity against methicillin-resistant *Staphylococcus aureus* (MRSA) (Farha et al. 2020). A synthetic cannabinoid HU-210 has been demonstrated to have inhibitory effect on quorum sensing (QS) and QS-dependent virulence properties in *Vibrio harveyi* (Soni et al. 2015). In addition, a recent study reported that CBD strongly inhibit the membrane vesicle formation in gram-negative bacteria and enhance the efficiency of bactericidal activity of antibiotics on both gram-positive and gram-negative bacteria (Kosgodage et al. 2019). These properties make cannabinoids as potential candidates for various applications including but not limited to inhibit dental plaque bacteria. Based on our preliminary observation, we recently reported the potential of cannabinoids on inhibiting the dental plaque bacteria (Stahl and Vasudevan 2020). We developed cannabinoids infused mouthwash products and our results in this study clearly demonstrate the efficacy of these mouthwash products against dental plaque bacteria.

Oral biofilm is a complex structure formed by sequential accumulation of over 600 species of bacteria (Aruni et al. 2015). The dental plaque includes, supragingival plaque and subgingival plaque. The supragingival plaque contains mainly of aerobic bacteria, in contrast to subgingival plaque which contains mostly of anaerobic bacteria (Sara et al. 2015). The accumulation of supragingival plaque eventually leads to the establishment of subgingival plaque. It has been suggested that supragingival plaque act as the reservoir of periodontal pathogens which potentially spread to and infect the subgingival sites (Do et al. 2013). Regular self-oral hygiene practices can effectively help to remove supragingival accumulation thereby suppresses periodontopathogens in subgingival plaque. Post-brushing rinsing/mouthwash helps to reduce dental plaque and gingivitis (Stoeken et al. 2007; Prasad et al. 2016). Most of the popular mouthwash products contain fluoride, CPC, alcohol or extreme pH. Fluoride retention in saliva after single use of fluoride containing mouthrinse (Mason et al. 2010) and erosion of dentin due to very low pH have been reported (Delgado et al. 2018). A comparative study involving 12 mouthrinse products and their effect on cytotoxicity and antimicrobial activity have reported heterogenous properties of mouthwash products and some of the popular mouthwash products had poor antimicrobial properties but higher cytotoxicity (Müller et al. 2017). Several studies have reported chlorhexidine as the only mouthwash to be very effective in controlling dental plaque (Zheng and Wang 2011; Richards 2015; Müller et al. 2017; Varghese et al. 2019). However, chlorhexidine causes unpleasant side effect of tooth surface discoloration. Addition of an antidiscoloration system with chlorhexidine might combat the side effect of tooth discoloration but only at the cost of loss of actual function of chlorhexidine. A controlled 3-weeks clinical trial involving 26 students reported that chlorhexidine in combination with antidiscoloration system did not prevent plaque or gingivitis development (Li et al. 2014). Another recent research report also concluded that addition of antidiscoloration system decreases the efficiency of chlorhexidine (Guerra et al. 2019). Although chlorhexidine is still the gold standard, it is suitable for short prescribed period as advised by the dentist and not suitable for regular use. Therefore, a safer and effective alternative is needed for regular use to maintain the oral hygiene. Several herbal mouthwashes were reported to have potential to replace chlorhexidine, however they lack sufficient scientific study and evidence (Dhingra and Vandana 2017; Al-Maweri et al. 2020). In this study we have clearly demonstrated the efficacy of cannabinoids infused MW with controlled in vitro study. Both CBD-MW and CBG-MW showed high efficacy similar to that of gold standard for almost all the samples studied. With natural key ingredients, it is very unlikely for the CannIBite MW (CBD-MW and CBG-MW) products to cause any discoloration. However, the CannIBite MW products need to be test for tooth discoloration effect in comparison to chlorhexidine.

An advantage of this study is the approach of antibacterial assay involving total bacterial content in the dental plaque directly sampled from participants. Most of the studies involving mouthwash products tested the effect on pure isolates of few anaerobic bacterial species for example *S. mutans*. Although it is useful information and evidence, it is not sufficient due to the fact that oral cavity is rich in diverse species of aerobic and anaerobic bacteria. Moreover, the oral biofilm varies from person to person. Also in this study we observed diversity in bacteria in culture plates irrespective of age, gender and DPSI score. Furthermore, considering that self-oral hygiene practices have effect mainly on the supragingival surface which contains mostly aerobic bacterial species, it is also important to test the efficiency of oral care products on culturable aerobic bacteria. Therefore, in this study we used the approach of evaluation against total-culturable aerobic bacteria from dental plaque samples. With this approach it is evident that CannIBIte mouthwash and chlorhexidine 0.2% are equally effective against total-culturable aerobic bacteria from dental plaque. It is also evident that even the most commonly available OTC-MW products tested in this study did not show appreciable bactericidal activity on total-culturable aerobic bacteria from dental plaque. Based on this study results, it will be interesting to study the in vivo performance of CannIBite mouthwash products in future to examine for other properties such as tooth discoloration and to examine the oral health benefits.

## Conclusion

Most of the reported studies show chlorhexidine containing mouthwash as the most effective mouthwash, however tooth staining is an unacceptable side effect of chlorhexidine. Therefore, it is not suitable for everyday or frequent use. Cannabinoids (CBD / CBG) infused mouthwashes together with other natural key ingredients shows promising bactericidal activity in vitro against total-culturable aerobic bacterial content in dental plaque, with efficiency equivalent to or better than that of the gold standard (0.2% chlorhexidine). CannIBite mouthwash products with cannabinoids infusion offer a safer and effective alternative without any fluorides or alcohol. Based on our in vitro study, the cannabinoids infused CannIBite mouthwash products offer a much safer, efficient and natural alternative to alcohol and/or fluoride containing mouthwashes.

## Supplementary information

**Additional file 1: Figure S1.** Comparison of DEV-Nutrient agar vs LB agar plate.

**Additional file 2: Figure S2.** Determination of minimum inhibitory concentration for CannIBite mouthwash products. In X-axis, 0 to 11 refers to serial dilution (0 represents no dilution). Y-axis represents approximate percentage of turbidity, which represents bacterial growth.

**Additional file 3.** Data on average of zone of inhibition measurements for test and control mouthwash products.

**Additional file 4.** Raw data on zone of inhibition measurements for test and control mouthwash products.

**Additional file 5.** Data on comparative statistical analyses (DPSI score, age, gender and product).

## Data Availability

Additional data are available as supplementary items. Any further details on results, data and methods can be requested directly to corresponding author.

## Abbreviations

CBD: Cannabidiol
CBG: Cannabigerol
CHX: Chlorhexidine digluconate 0.2%
CPC: Cetylpyridinium chloride
DPSI: Dutch periodontal screening index
MRSA: Methicillin-resistant *Staphylococcus aureus*
MW: Mouthwash
OTC: Over the counter
PBS: Phosphate buffer saline
THC: Δ9-tetrahydrocannabinol
