# Supplementary figures and images for: Cannabinoids infused mouthwash products are as effective as chlorhexidine on inhibition of total-culturable bacterial content in dental plaque samples
Source: J Cannabis Res. 2020 Jun 23;2:20. doi: 10.1186/s42238-020-00027-z (PMC7819473; doi:10.1186/s42238-020-00027-z)

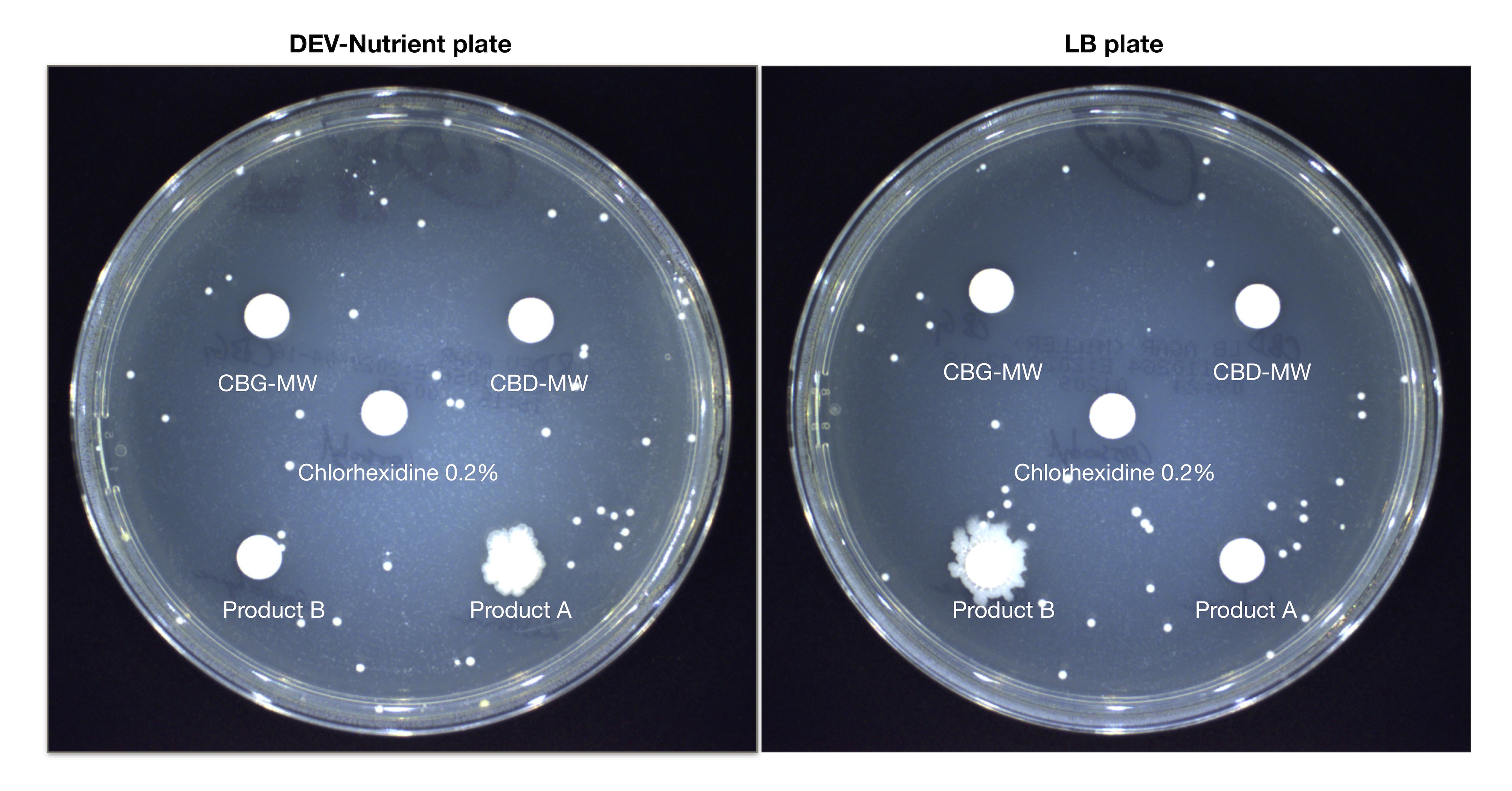

Supplement: Supplementary file 1 — Additional file 1: Figure S1. Comparison of DEV-Nutrient agar vs LB agar plate. [file 42238_2020_27_MOESM1_ESM.jpg]

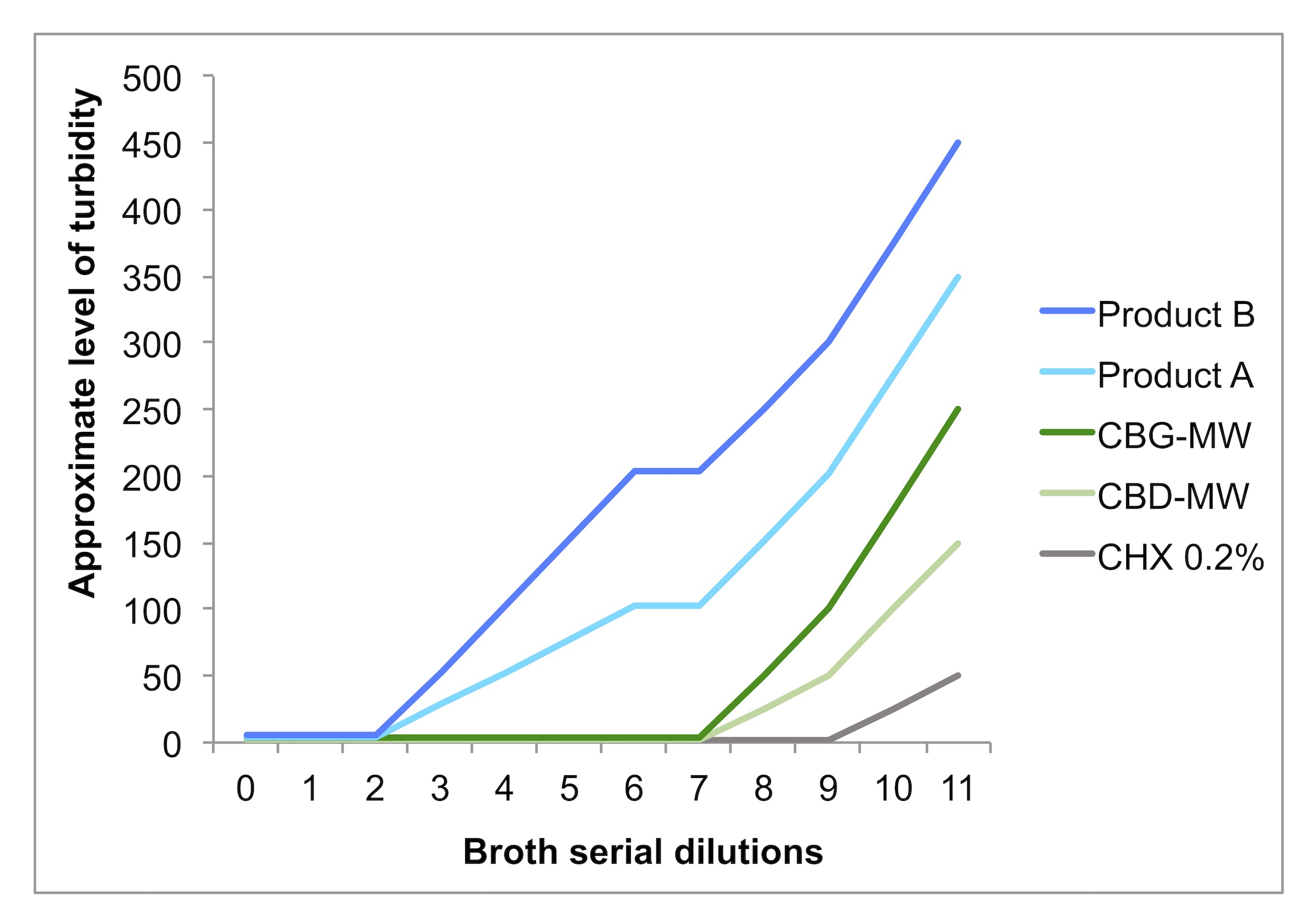

Supplement: Supplementary file 2 — Additional file 2: Figure S2. Determination of minimum inhibitory concentration for CannIBite mouthwash products. In X-axis, 0 to 11 refers to serial dilution (0 represents no dilution). Y-axis represents approximate percentage of turbidity, which represents bacterial growth. [file 42238_2020_27_MOESM2_ESM.jpg]
